# Distinct therapeutic effects of auraptene and umbelliprenin on TNF-α and IL-17 levels in a murine model of chronic inflammation

**DOI:** 10.1016/j.heliyon.2024.e40731

**Published:** 2024-11-26

**Authors:** Saeid Joveini, Fatemeh Yarmohammadi, Mehrdad Iranshahi, Amin Reza Nikpoor, Vahid Reza Askari, Armin Attaranzadeh, Leila Etemad, Zhila Taherzadeh

**Affiliations:** a Department of Pharmacodynamy and Toxicology, School of Pharmacy, Mashhad University of Medical Sciences, Mashhad, Iran; b Medical Biology Research Center, Health Technology Institute, Kermanshah University of Medical Sciences, Kermanshah, Iran; c Biotechnology Research Center, Pharmaceutical Technology Institute, Mashhad University of Medical Sciences, Mashhad, Iran; d Molecular Medicine Research Center, Hormozgan Health Institute, Hormozgan University of Medical Sciences, Bandar Abbas, Iran; e Pharmacological Research Center of Medicinal Plants, Mashhad University of Medical Sciences, Mashhad, Iran; f Department of Medical Genetics, Faculty of Medicines, Mashhad University of Medical Sciences, Mashhad, Iran; g Pharmaceutical Research Center, Pharmaceutical Technology Institute, Mashhad University of Medical Sciences, Mashhad, Iran; h Applied Biomedical Research Center, Mashhad University of Medical Sciences, Mashhad, Iran; i Targeted Drug Delivery Research Center, Mashhad University of Medical Sciences, Mashhad, Iran

**Keywords:** Aurapten, Umblliprenine, Rheumatoid arthritis, TNF-α, IL-17

## Abstract

**Objective:**

To compare the anti-arthritic potential of orally administered auraptene (AUR) and umbellliprenin (UMB) in chronic inflammation by exploring the differential effect on regulating TNF-α and IL-17.

**Methods & materials:**

Sixty male rats were divided into ten groups, and after confirming chronic inflammation, the treatment groups received AUR or UMB orally for 9 days. On day 16, histopathological changes were evaluated. Altered serum levels of the inflammatory cytokines TNF-α and IL-17 were examined as the underlying mechanisms.

**Results:**

Administering AUR orally at 16 mM/kg caused a significant increase in body weight gain compared to the baseline (p < 0.05), while UMB at a dose of 64 mM/kg significantly reduced edema size (p < 0.01). TNF-α levels were significantly lower in all doses of AUR and UMB treatments compared to the arthritis control group (p < 0.05). Treatment with AUR at all relative doses resulted in a significant decrease in IL-17 levels compared to the arthritis control group (p < 0.05), whereas UMB treatment did not show a significant effect on IL-17 levels.

**Conclusion:**

AUR and UMB regulate TNF-α and IL-17 differently; AUR inhibits both, showing broad therapeutic potential, while UMB specifically targets TNF-α, showing a specialized role.

## Highlights

• AUR (auraptene) and UMB (umbellliprenin) have distinct mechanisms of action in modulating TNF-α and IL-17 levels.
• AUR achieves its anti-inflammatory effect through the combined blockade of TNF-α and IL-17.
• UMB's anti-inflammatory effects are primarily attributed to its impact on TNF-α.
• These findings highlight the potential therapeutic applications of AUR and UMB in inflammatory diseases, emphasizing the importance of considering the combined blockade of TNF-α and IL-17 for more effective treatment strategies.

## Abbreviations

ANOVA: Analysis of variance
ARRIVE: Animal research: reporting of in vivo experiments
AUR: Auraptene
CFA: Complete Freund's adjuvant
DBU: 1, 8-diazabicyclo-(5.4.0)-undec-7-ene
EDTA: Ethylenediaminetetraacetic acid
ELISA: Enzyme-linked immunosorbent assay
H&E: Hematoxylin and eosin
IL: Interleukin
MS: Multiple Sclerosis
MUMS: Mashhad University of Medical Sciences
PBS: Phosphate-buffered saline
pg/L: Picogram per liter
PV-I: Perivascular infiltration
ROS: Reactive oxygen species
RNS: Reactive nitrogen species
SEM: Standard error of mean
Th17: T helper 17
TLC: Thin layer chromatography
TNF-α: Tumor necrosis factor-alpha
UMB: Umblliprenine

## 1 Introduction

Inflammatory diseases represent a diverse group of weakening conditions that arise from dysregulated immune responses, leading to tissue damage and chronic inflammation [[1], [2], [3]]. Among the various cytokines involved in the complex network of inflammatory processes, tumor necrosis factor-alpha (TNF-α) and interleukin (IL (-17 have emerged as key players, playing critical roles in the pathogenesis of various inflammatory disorders [[2], [3], [4], [5], [6], [7]]. As a result, targeting these cytokines has emerged as a promising strategy for developing therapeutic interventions aimed at reducing inflammation and its related complications. TNF-α, a pro-inflammatory cytokine mainly produced by activated macrophages and T cells, plays a crucial role in coordinating a series of immune responses during inflammation [[8], [9], [10], [11], [12]]. Some of its functions include the activation of immune cells, the activation of adhesion molecules on endothelial cells, and the induction of other pro-inflammatory cytokines [[11], [12], [13]]. Imbalanced TNF-α production in some patients has been accepted as having associations with a range of inflammatory diseases, such as rheumatoid arthritis, inflammatory bowel disease, psoriasis and systemic lupus erythematosus in some patients [10,11]. TNF-α stimulates the secretion of metalloproteinases, enzymes which degrade surrounding tissues in cases of arthritis [14]. TNF-α is the only cytokine for which there is a correlation between high levels in the cells and disease stage, mentioning its fundamentals as a therapeutic target [12]. Th17 cells are the main source of IL-17, which is an important factor for coordinating the immune responses [3,[15], [16], [17], [18], [19], [20]]. IL-17 also supports neutrophil recruitment and activation, enhances the production of pro-inflammatory cytokines and chemokines, and leads to tissue inflammation and injury [3,14,[16], [17], [18], [19],^21^]. IL-17 signaling abnormalities have been not only confined to psoriasis, multiple sclerosis and rheumatoid arthritis, they have also been linked to other inflammatory diseases of the lungs [3,15,18,[22], [23], [24]]. Given the critical roles of TNF-α and IL-17 in driving inflammatory responses, their careful regulation is essential for restoring immune balance and improving inflammatory conditions [[3], [4], [5], [6],^18^,^19^]. Several clinical studies on the targeted treatment of patients with high levels of TNF-a and IL-17 have also shaped pleasing results [3,15,18,19,25,26]. Biologics, such as TNF-α inhibitors and IL-17 antagonists, have transformed the management of several inflammatory disorders [3,15,18,19,[25], [26], [27]].

In an inflammatory condition, a resident cell can also be an immune cell that induces the release of reactive oxygen species (ROS) which results in oxidative stress, and there is a close association between oxidative stress and inflammation [28]. The natural compounds such as AUR and UMB, which have been shown to stress that possess anti-oxidative properties, are natural products registered as being of anti-inflammation [29,30]. These compounds have been isolated from plants belonging to the family of Rutaceae. The family has a variety of plants that possess medicinal values beneficial to human species [30,31].

In our previous study, the researchers used various doses of AUR and UMB as protective agents in an experimental chronic inflammatory model induced by complete Freund's adjuvant (CFA) in rats [32]. In fact, that study showed that AUR and UMB could prevent inflammation through the reduction of the IL-17 serum level, and thus may be promising drugs in the treatment of inflammatory diseases [32]. The authors of that earlier study concluded that the possible use of AUR and UMB in inflammatory diseases may vary because of their different cytokine release and inhibition patterns [32]. Given that cytokine modulation has promising therapeutic potential [3,12,25,27,32], in this research, the aim was to study the influence of two natural compounds, AUR and UMB, on TNF-α and IL-17 levels in an experimental chronic inflammatory model. By pointing out the possible efficacy of these compounds in the modulation of TNF-α and IL-17, the present study thus provides new possible therapeutic approaches for the management of inflammatory diseases. The actual mechanisms by which AUR and UMB modulate these cytokines in this experimental model of chronic inflammation will better explain the development of targeted, effective treatment strategies.

## 2 Materials and methods

### 2.1 Synthesis and characterization of AUR and UMB

AUR and UMB were synthesized using previously described methods [32,33]. Briefly, AUR and UMB were synthesized by reacting 72.89 g of hydroxycoumarin with 5 g of geranyl bromide (for AUR) or 5 g of farnesyl bromide (for UMB) in acetone, along with 20 g of an alkaline substance, such as potassium carbonate, sodium carbonate, or 1,8-diazabicyclo-(5.4.0)-undec-7-ene (DBU), at room temperature for approximately 12 h of reflux. The solution was then filtered, and thin-layer chromatography (TLC) was used to remove impurities. Next, the product was dried under a hood, and dichloromethane was gradually added until the AUR was fully dissolved. To separate the AUR, the solution was filtered, and methanol was subsequently mixed with the filtrate. Under a hood, the solution was dried until the dichloromethane evaporated, leading to the crystallization and separation of the AUR from the methanol. Confirmation of the product was achieved by analyzing NMR, melting point, and TLC spectra. The melting point of AUR was found to be in the range of 64.2–67.9 °C, while the melting point of UMB was 55–60 °C, which is acceptable.

### 2.2 Animals and ethics statement

Sixty adult male Wistar rats, aged 6–8 weeks, with a mean weight of 180–200 g, were obtained from the Animal House, School of Pharmacy, Mashhad University of Medical Sciences, Mashhad, Iran. The rats were housed in a controlled environment with a standard temperature of 22 ± 4 °C and humidity, under a cycle of 12:12 h light/dark, with free access to food and water. All experiments were conducted under the Animal Research: Reporting of In Vivo Experiments (ARRIVE) guidelines and regulations [34]. The experimental protocols were approved by a local animal care, following the eighth edition of the Guide for the Care and Use of Laboratory Animals [35], with the committee's approval number being IR. MUMS. sp.1394.5.

### 2.3 Induction of chronic inflammation arthritis

Male Wistar rats were anesthetized with ketamine/xylazine (100/10 mg/kg, IP) [36]. Chronic inflammatory arthritis was induced by a single subcutaneous injection of 100 μl of CFA (heat-killed *Mycobacterium tuberculosis* in paraffin oil, 0.5 mg/ml) into their right sole. CFA induces an enhanced immune response, resulting in localized redness and swelling, and induces inflammation 3 h after injection, which may persist for a duration of up to four weeks [37]. The control group was not subjected to any injection. As part of the inflammatory experiment, an internal control for arthritis was established in all injected rats by administering a single subcutaneous injection (0.1 ml) of phosphate-buffered saline (PBS) to the left hind foot pads, without the presence of any inflammation-inducing agent. The correct injection of CFA was confirmed to control the size and eliminate the effect of nonspecific inflammation.

### 2.4 Experimental design and treatment administration

Following an accommodation period, the animals were randomly assigned to the following ten groups (n = 6):

1. Arthritis control group in which the immunization of rats is performed only by injection of CFA, but the rats did not receive any treatment.
2. AUR-treatment group 1 received AUR daily (4.77 mg/kg/day or 16 mM/kg).
3. AUR-treatment group 2 received AUR daily (9.53 mg/kg/day or 32 mM/kg).
4. AUR-treatment group 3 received AUR daily (19 mg/kg/day or 64 mM/kg).
5. UMB-treatment group 1 received UMB (5.85 mg/kg/day or 16 mM/kg).
6. UMB-treatment group 2 received UMB (11.7 mg/kg/day or 32 mM/kg).
7. UMB-treatment group 3 received UMB (23.4 mg/kg/day or 64 mM/kg).
8. Positive control group 1 included treatment with indomethacin as a standard and non-steroidal anti-inflammatory drug (3 mg/kg/day or 8 mM/kg).
9. Positive control group 2 included therapy with prednisolone as standard and steroidal anti-inflammatory drugs (5 mg/kg/day or 8 mM/kg).
10. Normal control group in which healthy rats received only normal saline (with no inflammation-inducing agent).

On the 7th day after the onset of chronic inflammation, the oral administration of AUR and UMB was started and sustained for a duration of nine days. Research has showed that the hindfoot injection of CFA, given as a single subcutaneous dose, can effectively induce a lesion that mimics rheumatoid arthritis in rats within a one-week period [38]. The doses used in this study were selected based on data from previous animal studies regarding the anti-inflammatory effects of AUR and UMB [[39], [40], [41]].

### 2.5 Clinical symptoms assessment

Clinical symptoms of the rats were assessed throughout the study period. This included monitoring the following parameters:

• Rats' body weight was assessed on a daily basis. The formula used to calculate the percentage change in measurements over a 16-day treatment period compared the values to the baseline (day 0) for each experimental group.

Normalized Weight Change =(Weight on Day (n)−Weight on Day (0)/Weight on Day (0)) × 100

• Local Adjuvant-induced Paw Edema Assessment: To evaluate the severity of the inflammatory arthritis model, hind paw swelling was measured in each rat. Measurements were taken just before the injection and throughout the 16-day period using a digital caliper [32]. The increased paw thickness for each rat was calculated as the difference between the left and right paw thickness and presented as the mean increase in paw thickness (mm). The ability of AUR and UMB to suppress paw inflammation was assessed by calculating the percentage of inhibition of paw edema, determined using the following formula [42].

$$\frac{\left(\text{Average}\;\text{increase}\;\text{in}\;\text{foot}-\text{pad}\;\text{thickness}\right){\;}_{\text{treatment}\;\text{group}}\;–\;{\left(\text{Average}\;\text{increase}\;\text{in}\;\text{foot}-\text{pad}\;\text{thickness}\right)\;}_{\text{arthritis}\;\text{control}\;\text{group}}}{{\left(\text{Average}\;\text{increase}\;\text{in}\;\text{foot}-\text{pad}\;\text{thickness}\right)\;}_{\text{arthritis}\;\text{control}\;\text{group}}}\times 100$$

The quantification of inflammation elimination was determined by evaluating the percentage change in paw edema, indicating whether the thickness decreased, increased, or remained the same compared to the initial condition [43].

### 2.6 Systemic evaluation of adjuvant-induced arthritis

At the end of the experiment, a systemic evaluation of adjuvant-induced arthritis was conducted. To prevent coagulation, blood samples were obtained via cardiac puncture and promptly transferred into tubes containing 10 mM ethylenediaminetetraacetic acid (EDTA). The blood samples were diluted and then cleansed to remove any impurities. The collected blood samples were centrifuged for 15 min at 2000 g to separate the serum. After isolation, the sera was quickly frozen and stored at −70 °C until analysis. Enzyme-linked immunosorbent assay (ELISA) kits from Eastbiopharm (Hangzhou, China) were employed to measure the levels of TNF-α and IL-17 in the serum samples, as per the manufacturer's instructions.

### 2.7 Histological assessment

After the animals were sacrificed, a histopathological examination was conducted to assess the extent of histopathological changes at the CFA injection site. A five mm punch biopsy was taken from the skin at the CFA injection site to obtain tissue samples for histopathological analysis. The samples were stored in 10 % neutral-buffered formalin for fixation. The tissue samples were processed and stained with hematoxylin & eosin (H&E), a commonly used staining method in histopathology. H&E staining allows for the visualization of cellular structures and identification of histopathological features.

A pathologist, blinded to the experimental groups, evaluated the histopathological features of skin inflammation. The evaluation encompassed the assessment of parameters, including inflammatory cell infiltration, intensity, granuloma formation, and keratinization. An analysis at the microscopic level was conducted to evaluate the infiltration of inflammatory cells in the interstitial and perivascular regions, noting any irregularities. The grading of the inflammatory process was determined by counting the number of inflammatory cells observed under a 400 × magnification. The grading criteria were categorized into three levels: mild, defined as fewer than ten inflammatory cells per field; moderate, characterized by the presence of 10–30 inflammatory cells per field; and severe, indicated by the presence of over 30 inflammatory cells per field [[44], [45], [46]].

The histopathological examination also included an assessment of granulomas, which are dense formations comprised epithelioid macrophages, as well as hyperkeratinization, characterized by excessive keratin formation and accumulation [47].

### 2.8 Statistical analysis

Data analysis was performed using GraphPad Prism (Version 6.01) software, and the results were reported as mean ± standard error of mean (SEM). Intra-group assessment was conducted using One-way repeated measures analysis of variance (ANOVA) to analyze the data during treatment, including sole size. Two-way ANOVA with Dunnett's *post hoc* test were used to compare the results between different groups. One-way ANOVA was used to compare the results among different groups at the end of the treatment.

The significance level for all tests was set at p ≤ 0.05. The results were reported using appropriate statistical symbols, such as asterisks, to show the level of significance.

## 3 Results

### 3.1 Impact of AUR and UMB on clinical symptom of adjuvant-induced chronic arthritis

One-Way ANOVA followed by Tukey's post hoc test showed significant differences in body weight changes across the various treatment groups (P < 0.0001) (Table 1). Specifically, notable improvements were seen in the higher concentrations of AUR (64 mM) and UMB (16 mM) when compared to the arthritis control group, with P-values of <0.0001 and 0.0127, respectively (Table 1). In addition, prednisolone showed a high effectiveness compared with the arthritis control group. Low-concentration treatments comprising AUR at 16 mM and UMB at 32 mM concentration did not show a significant difference from the control, hence less effective at that concentration, as depicted in Table 1.

**Table 1:** Effect of AUR and UMB on body weight changes over 16 days treatment.

Treatment groups	Weight changes % (n = 6)	P value
Arthritis control	0.99 ± 0.43	g
AUR 16 mM	0.32 ± 0.51	i
AUR 32 mM	3.03 ± 0.48	
AUR 64 mM	5.92 ± 0.70	c,f
UMB 16 mM	3.90 ± 0.59	a
UMB 32 mM	2.95 ± 0.54	
UMB 64 mM	3.34 ± 0.43	
Indomethacin 8 mM	2.31 ± 0.49	c
Prednisolone 8 mM	4.47 ± 0.73	
Normal control	5.47 ± 0.63	c

Results are expressed as Mean ± SEM and present the mean percentage changes in body weight observed over a 16-day period. Each entry reflects the average percentage change calculated from individual measurements, providing insights into weight fluctuations during the observation period. The statistical differences between groups were compared by one-way ANOVA with Tukey's multiple comparisons post-hoc test. ^a^ p < 0.05, ^b^p < 0.01, ^c^ p < 0.001 vs. arthritis control; ^d^ p < 0.05, ^e^ p < 0.01, ^f^ p < 0.001 vs. prednisolone-treated group; ^g^ p < 0.05, ^h^p < 0.01, ^i^ p < 0.001 vs. indomethacin -treated group.

**Fig. 1:**
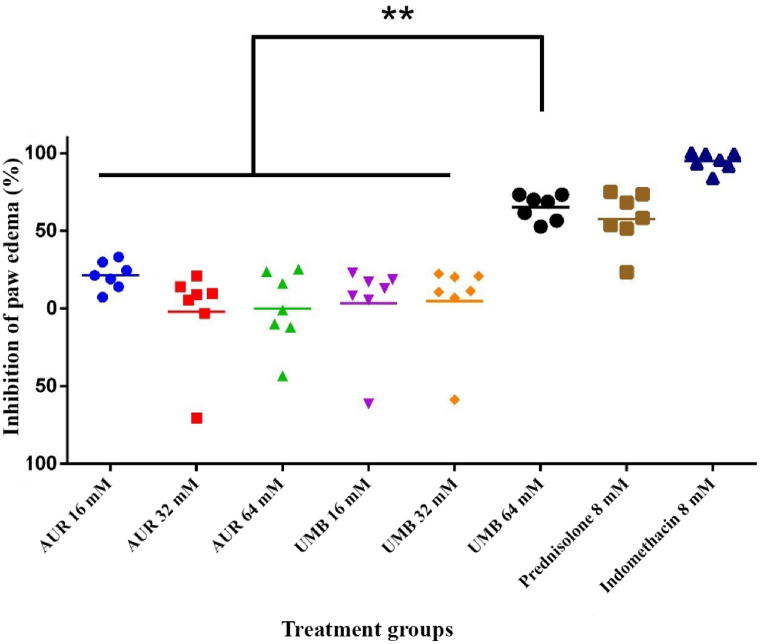
The potential of AUR and UMB to the inhibition of paw swelling on the adjuvant-induced chronic arthritis. Data are expressed as mean ± SEM; (n = 6). ∗∗p < 0.01 vs arthritis control group; repeated measures ANOVA with Dunnett's multiple comparisons *post-hoc* test.

Anti-inflammatory properties of AUR and UMB in adjuvant-induced chronic arthritis were studied based on their possible inhibition of swelling in the paws. According to Fig. 1, the data shows that the suppression of paw swelling varies from 6 % to 65 %. The highest overall percentage of inhibition can be seen in the groups treated with 64 mM UMB, at 65.3 ± 7.6 %. The results showed that at doses where oral AUR administration resulted in the inhibition of paw swelling by 0–21 %, the highest was recorded for the dose of 16 mM with a percentage of 21 %. Supporting this statement, the high percentage of inflammation inhibition-94.9 ± 5.4%-was further observed following the administration of indomethacin (8 mM) to the rat model of chronic inflammation arthritis compared to the arthritis control group, at p < 0.01. The greater inhibition of swelling by UMB 64 mM and indomethacin 8 mM was superior to the other compounds tested in terms of anti-inflammatory effects.

### 3.2 Impact of AUR and UMB on TNF-α and IL-17 levels in adjuvant-induced chronic arthritis

**Fig. 2:**
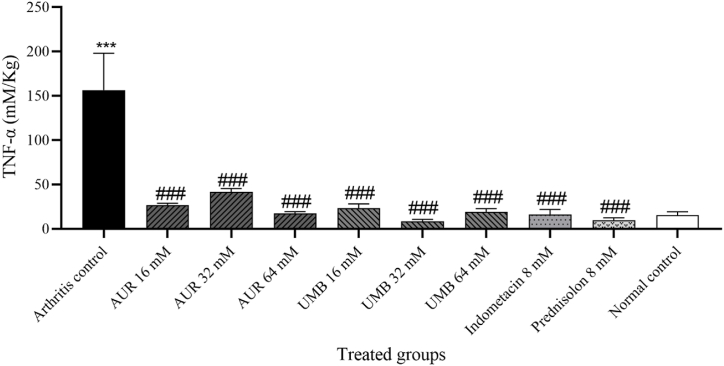
Effects of AUR and UMB on TNF-α serum level. The statistical differences between groups were compared by one-way ANOVA and Bonferroni's Post Hoc Test. Statistical significance was considered at p < 0.05. Experimental data were expressed as the mean ± SEM. ∗∗∗p < 0.001 compared to the normal control group. ###p < 0.001 compared with the arthritis control group.

Importantly, post adjuvant injection, levels of TNF-α were also significantly higher in the arthritis control group compared with the normal control group (15.6 ± 3.7 pg/L and 156.6 ± 41.3 pg/L, respectively) (p < 0.001), confirming successful induction of the inflammatory response (Fig. 2).

However, the AUR and UMB treatments revealed a significant decline in the levels of TNF-α for all of their dosages when compared to the arthritis control, with p < 0.001, reflecting their potent anti-inflammatory properties, as shown in Fig. 2. As expected, the positive controls, treated either with prednisolone or indomethacin, showed significantly reduced TNF-α levels regarding the arthritis control group, p < 0.001, evidencing drug efficacy in the repression of the inflammatory response elicited by adjuvant injection (Fig. 2).

With other treatments, the lowest mean TNF-α levels were for the treatment groups of UMB at 32 mM, 8.142 ± 2.4 pg/L, and prednisolone at 8 mM, 9.55 ± 2.8 pg/L.

**Fig. 3:**
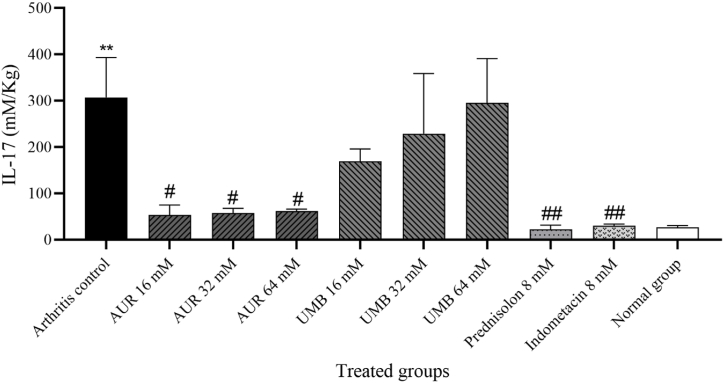
Effects of AUR and UMB on IL-17 serum level. The statistical differences between groups were compared by one-way ANOVA and Bonferroni's Post Hoc Test. Statistical significance was considered at p < 0.05. Experimental data were expressed as the mean ± SEM. ∗∗p < 0.01 compared to the normal control group. #p < 0.05 and ##p < 0.01 compared with the arthritis control group.

Compared with the normal control group, there was a significant increase of IL-17 levels in the arthritis control group: 307.29 ± 86.37 pg/L versus 26.37 ± 4.4 pg/L, p < 0.01, as represented in Fig. 3. AUR, in all of its relative doses, presented a significant reduction of IL-17 levels compared to the arthritis control group. The p-value was less than 0.05, as represented in Fig. 3. On the other hand, UMB treatment was not significantly different from the arthritis control in the variation of IL-17 levels; hence, UMB may not be a strong modulator in this model regarding IL-17 variation (Fig. 3). The values of IL-17 obtained from the lowest levels were 8 mM with a means of 22.51 ± 8.8 pg/L in the prednisolone- and 29.93 ± 4.1 pg/L in the indomethacin-treated group; this thus showed anti-inflammatory roles or suppression of the production of IL-17 (Fig. 3).

### 3.3 Histopathological evaluations

**Fig. 4:**
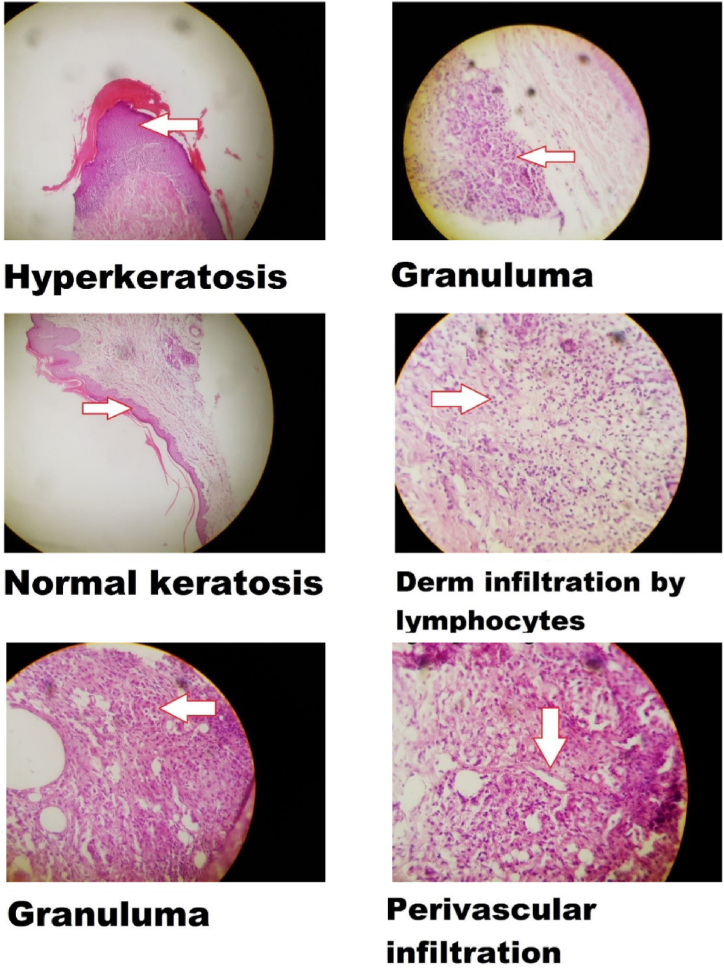
Representative pictures of skin sections stained with hematoxylin and eosin (H&E) examined under a microscope (magnification × 400).

The precisely prepared tissue sections revealed discernible characteristics, which were categorized and quantified, as outlined in both Table 2 and Fig. 4. These features encompassed granulomas, a manifestation of chronic inflammation; keratosis, characterized by excessive cornification of the epidermis; infiltration, indicative of an immune response involving mononuclear cells such as lymphocytes and macrophages; and fibrosis, the aberrant accumulation of extracellular matrix components within the tissue microenvironment.

**Table 2:** Histopathological analysis of skin sections over 16 days.

Treatment groups	Intensity	Granulomas	Keratosis	Inflammatory cell infiltration
**Arthritis control**	Sever	∗	↑↑	PV-I
**AUR 16 mM**	Sever	∗	↑↑	I
**AUR 32 mM**	Sever	∗	↑↑	I
**AUR 64 mM**	Sever	∗	↑	I
**UMB 16 mM**	Sever	∗	↑	I
**UMB 32 mM**	Sever	∗	↑	I
**UMB 64 mM**	Moderate	∗	↑↓	I
**Indomethacin 8 mM**	Moderate	∗	↑	I
**Prednisolone 8 mM**	Moderate	∗	↑	I
**Normal control**	Mild		↑↓	I

Mild: N ≤ 10, Moderate:10 ≤ N ≤ 30 Infiltration, Sever: 30 ≤ N ≤ 50. ∗: The presence of granulomas in the tissue samples, ↑:Increased keratosis compared to normal skin, ↓:Decreased keratosis compared to normal skin, ↑↓: The level of keratosis is not different from normal skin, I: Presence of inflammatory cells in the tissue samples, PV-I: Perivascular Infiltration.

Severe inflammatory cell infiltration was observed surrounding the arteries in the hind paws of rats sixteen days following the administration of CFA and seven days after the initiation of treatment. The group receiving 64 mM/kg of UMB showed the least amount of inflammatory cell infiltration, similar to the groups taking prednisolone or indomethacin. Subcutaneous injection of CFA resulted in granuloma formation compared with the normal control group. No treatment could prevent granuloma formation in mice injected with CFA. The highest rates of keratosis were observed in the 16 mM/kg and 32 mM/kg AUR groups, respectively. Although interstitial infiltration occurred in all groups after CFA exposure, perivascular infiltration was observed only in the arthritis control group.

## 4 Discussion

Our study demonstrates a significant increase in TNF-α and IL-17 levels following the administration of an adjuvant, along with a marked reduction in TNF-α levels upon exposure to AUR and UMB. These two pharmacological agents, currently under investigation, demonstrated divergent effects on the levels of IL-17 within the established model of chronic inflammation. Although AUR effectively reduced IL-17 levels, the use of UMB did not show a significant impact on IL-17 modulation under the same experimental conditions.

Upon conducting a thorough histological examination, we discovered that the paw tissue of the untreated control groups in the chronic inflammatory model was marked by severe localized inflammation. This pathological state was characterized by pronounced hyperplasia, substantial infiltration of inflammatory cells, elevated cellular proliferation rates, and the presence of keratosis, as precisely detailed in Table 1. The notable exception to this was the UMB treatment administered at a concentration of 64 mM (equivalent to 23.4 mg/kg/day), which exhibited a significant reduction in the magnitude of the inflammatory response. This observation underscores the potent anti-arthritic properties of UMB at higher dosages.

The increasing academic interest in AUR and UMB can be credited to their proven ability to regulate the complex immune response system. Preliminary studies, including the ones referenced here [31,32,[48], [49], [50]], suggest that these substances may exert their anti-inflammatory effects by altering specific patterns of cytokine release and inhibition. Our experimental results indicate that these two agents may have separate mechanisms or differing levels of involvement in the intricate biochemical pathways associated with inflammation. It is crucial to possess a thorough comprehension of the mechanisms of each treatment, as this knowledge can influence their effectiveness and the most suitable dosage schedules for arthritis treatment.

Inflammation can contribute to the production of reactive oxygen and nitrogen species (ROS and RNS), further exacerbating oxidative stress and creating a vicious circle that promotes the development of pathophysiological mechanisms in diseases like multiple sclerosis (MS) [51]. Antioxidants have shown therapeutic potential in mitigating the negative impact of oxidative stress and inflammation on health [52]. AUR and UMB are natural compounds that have been shown to possess anti-inflammatory effects, which may be attributed to their antioxidant properties [31,53].

IL-17and TNF-α are two key pro-inflammatory cytokines that play critical roles in the pathogenesis of various inflammatory and autoimmune diseases, including arthritis [14,17,54]. When IL-17 and TNF-α are present together, they synergize to enhance the expression of inflammatory genes and promote a more robust and sustained inflammatory response [14,55,56]. This cooperation between IL-17 and TNF-α creates a positive feedback loop, amplifying the pro-inflammatory signaling and contributing to the pathogenesis of chronic inflammatory conditions like arthritis [5,14].

We showed AUR exhibits the ability to target both IL-17 and TNF-α pathways, making it a promising candidate for simultaneously targeting multiple pro-inflammatory pathways. This suggests that AUR could be a potential therapeutic option for chronic inflammatory diseases [32]. In contrast, the anti-inflammatory effects of UMB may not be primarily linked to IL-17 pathways in this specific context. However, it is important to note that UMB treatment led to a reduction in TNF-α levels, showing that its anti-inflammatory mechanism may be primarily associated with targeting TNF-α production rather than IL-17 [57]. Therefore, UMB may also be a potential therapeutic option for chronic inflammatory diseases due to its ability to improve tissue injury and reduce TNF-α levels.

Clinical studies have showed that blocking either IL-17 or TNF-α individually can lead to some improvement in arthritis symptoms [3,6,58]. However, since IL-17 and TNF-α synergize to amplify inflammation, the combined blockade of both cytokines has been shown to have a more potent therapeutic effect, leading to greater disease amelioration [54,59]. Combining anti-IL-17 and anti-TNF-α therapies can target different pathways of inflammation, disrupting the synergistic signaling between these cytokines. This combination therapy can lead to better control of inflammation, a reduction in disease activity, and improvements in clinical outcomes for patients with arthritis. Combined blockade may also reduce the risk of developing resistance to treatment, as targeting multiple pathways simultaneously reduces the likelihood of compensatory mechanisms emerging [14,59].

In our study, regarding the combined blockade of TNF-α and IL-17, AUR showed a more significant effect than UMB in reducing both cytokines. This suggests that AUR may be a more effective treatment option for blocking both TNF-α and IL-17.

The timing of administration of AUR and UMB may impact influence their effects on TNF-α and IL-17 levels. In cases of adjuvant-induced arthritis, administration of AUR and UMB concurrently with the induction of arthritis showed the ability to reduce serum IL-17 levels, while UMB treatment alone exhibited the capacity to suppress local inflammation [32].

Motivated by these encouraging preliminary findings, the present research sought to delve deeper into the therapeutic potential of AUR and UMB in a more clinically relevant setting. Instead of administering these agents concurrently with CFA induction, we aimed to investigate the impact of orally administering AUR and UMB one week after Adjuvant-induced chronic inflammation. This delayed treatment approach is more representative of the clinical scenario, where patients are often diagnosed at later stages of RA progression.

In the present study, we showed that in the same model of established adjuvant-induced arthritis, a one-week delay in oral AUR administration resulted in reductions in both TNF-α and IL-17 levels. In contrast, UMB treatment did not significantly impact IL-17 modulation but prove to be more effective in suppressing local inflammation. The geranyl and farnesyl groups present in these compounds may be responsible for their distinct anti-inflammatory effects by allowing for their interaction with proteins involved in inflammatory signaling pathways, ultimately leading to the inhibition of these pathways and a reduction in inflammation [60].

When comparing the effects of AUR and UMB, they exert distinct effects on both inflammation and cytokine levels. These findings indicate that AUR and UMB may target different components of the inflammatory response, with UMB potentially influencing early inflammatory processes, while AUR may be more effective in managing ongoing inflammation by regulating key pro-inflammatory cytokines like TNF-α and IL-17.

## 5 Conclusion

AUR and UMB exhibit distinct mechanisms in modulating the levels of TNF-α and IL-17, key pro-inflammatory cytokines implicated in the pathophysiology of various inflammatory disorders. AUR achieves its anti-inflammatory properties through a dual-mode approach, encompassing the inhibition of both TNF-α and IL-17, thereby underscoring its potential as a versatile therapeutic agent capable of simultaneously targeting two critical inflammatory mediators. In contrast, UMB's primary modulatory influence is focused on TNF-α, suggesting a more specialized role in controlling the inflammatory milieu. These observations underscore the therapeutic implications of employing AUR and UMB in the treatment of inflammatory pathologies, with a particular emphasis on the potential efficacy of strategies that encompass the simultaneous inhibition of TNF-α and IL-17. To fully appreciate and optimize the therapeutic potential of these compounds, further comprehensive research is warranted to explain the precise molecular and cellular mechanisms by which AUR and UMB exert their effects, thereby facilitating the informed design and implementation of clinical studies that can harness their anti-inflammatory properties in a thoughtful and effective manner.

## CRediT authorship contribution statement

**Saeid Joveini:** Writing – review & editing, Writing – original draft, Visualization, Validation, Investigation, Formal analysis, Data curation. **Fatemeh Yarmohammadi:** Writing – review & editing, Writing – original draft, Visualization, Software, Formal analysis, Data curation. **Mehrdad Iranshahi:** Writing – review & editing, Writing – original draft, Validation, Supervision, Resources, Funding acquisition, Formal analysis. **Amin Reza Nikpoor:** Writing – review & editing, Writing – original draft, Visualization, Validation, Software, Investigation, Formal analysis, Data curation. **Vahid Reza Askari:** Writing – review & editing, Writing – original draft, Visualization, Supervision, Methodology, Formal analysis, Data curation. **Armin Attaranzadeh:** Writing – review & editing, Writing – original draft, Visualization, Supervision, Investigation, Formal analysis, Data curation. **Leila Etemad:** Writing – review & editing, Writing – original draft, Visualization, Software, Formal analysis, Data curation. **Zhila Taherzadeh:** Writing – review & editing, Writing – original draft, Validation, Supervision, Resources, Project administration, Methodology, Funding acquisition, Conceptualization.

## Data availability

The data supporting the findings of this study are available upon reasonable request from the corresponding author, as they are not publicly accessible due to privacy concerns.

## Declaration of generative AI and AI-assisted technologies in the writing process

In the course of developing this manuscript, the authors utilized Grammarly to enhance the language and clarity of the text. Following the use of this tool, the authors carefully reviewed and revised the content as necessary, assuming complete responsibility for the final publication.

## Funding

The financial backing for this research was provided by the Vice-Chancellor for Research at 10.13039/501100004748Mashhad University of Medical Sciences in Mashhad, Iran.

## Declaration of competing interest

The authors declare that they have no known competing financial interests or personal relationships that could have appeared to influence the work reported in this paper.
